# Better Safe than Sorry: Rheumatoid Arthritis, Interstitial Lung Disease, and Medication—A Narrative Review

**DOI:** 10.3390/biomedicines11061755

**Published:** 2023-06-19

**Authors:** Iulia-Tania Andronache, Victoria-Cristina Şuţa, Maria Şuţa, Sabina-Livia Ciocodei, Liliana Vladareanu, Alina Doina Nicoara, Oana Cristina Arghir

**Affiliations:** 1 Doctoral School of Medicine, “Ovidius” University of Constanta, 900470 Constanta, Romania; maria_suta@yahoo.com (M.Ş.); arghir_oana@yahoo.com (O.C.A.); 2 Department of Rheumatology, Internal Medicine Clinic, “Dr. Alexandru Gafencu” Military Emergency Hospital Constanta, 900527 Constanta, Romania; 3 3rd Department—1st Clinical Medical Disciplines, Faculty of Medicine, “Ovidius” University of Constanta, 900470 Constanta, Romania; 4 4th Department—2nd Clinical Medical Disciplines, Faculty of Medicine, “Ovidius” University of Constanta, 900470 Constanta, Romania

**Keywords:** rheumatoid arthritis, drug-induced lung injury, interstitial lung disease, pulmonary fibrosis

## Abstract

It is well known that rheumatoid arthritis (RA) patients are at an increased risk of developing non-infectious pulmonary complications, especially interstitial lung disease (ILD); however, the clinician must keep in mind that lung disease could not only be a manifestation of the underlying condition, but also a consequence of using disease-modifying therapies. New-onset ILD or ILD worsening has also been reported as a possible consequence of both conventional disease-modifying antirheumatic drugs (DMARDs) and biologic agents. This study is a narrative review of the current literature regarding the potential risk of developing interstitial lung disease along with the administration of specific drugs used in controlling rheumatoid arthritis. Its purpose is to fill knowledge gaps related to this challenging patient cohort by addressing various aspects of the disease, including prevalence, disease features, treatment strategies, and patient outcomes.

## 1. Introduction

Drug-induced lung injury (DLI) is defined as a lung injury that specifically results from the use of a drug, including prescription and over-the-counter drugs, herbal medicines, supplements, and illegal narcotics [1].

As causality is often difficult to prove, attributing lung injury to medication is a challenging task. The diagnosis is usually presumptive, lacking a gold standard test, and the causal connection is determined by the temporal association between drug initiation, symptom onset, radiologic abnormalities, the failure to identify a microbial agent, the absence of pre-existing lung disease, and improvement after drug discontinuation [1,2].

Based on these principles, the 2013 Consensus statement for the diagnosis and treatment of drug-induced lung injuries recommends the following criteria for drug-induced lung injury [1,3,4] (Table 1).

Although the exact pathogenic mechanisms of drug-induced lung injury (DLI) have not been fully understood, except for a limited number of drugs, two potential primary mechanisms have been proposed: the cytotoxic and the immune mechanisms of action. These mechanisms may be independently involved or in combination, resulting in various forms of lung injury. These two mechanisms may be influenced by contextual factors, such as smoking or underlying lung diseases (Table 2) [1].

Whilst the use of biologic disease-modifying agents to treat RA has led to an unprecedented improvement in clinical and functional outcomes, their safety profile remains to be fully elucidated. Almost all DMARDs are known to potentially trigger DLI in patients with RA [1,5,6]. A very useful tool when considering DLI is The Drug-Induced Respiratory Disease Website developed by Philippe Camus—http://www.pneumotox.com (accessed on 10 April 2023). This website is a simple, easy-to-use tool, in which, after introducing the generic name of the drug, a frequency or incidence gauge (in the form of digits within stars) indicates how many cases have been published in the literature (questionable signal; 1 <10 cases; 2 10–50 cases; 3 50–100 cases; 4 100–200 cases; 5 >200 cases) [5]. The website also provides abundant links to the existing literature and case reports.

The treat-to-target (T2T) strategy in rheumatoid arthritis (RA) is an approach that involves setting a treatment goal (RA remission or low disease activity) based on disease activity scores [7]. This strategy involves early aggressive treatment with one or more conventional synthetic DMARDs and/or biologic DMARDs/targeted—tsDMARDs (Table 3), along with symptomatic therapy that may include NSAIDs, low-dose prednisone, and physical therapy.

## 2. Conventional DMARDS

### 2.1. Methotrexate 

By inhibiting the enzyme AICAR transformylase, Methotrexate disrupts the metabolism of adenosine and guanine, resulting in the accumulation of adenosine. The increased levels of adenosine contribute to the anti-inflammatory effects by suppressing T-cell activation, down-regulating B-cells, and enhancing the sensitivity of activated CD-95 T cells. [5,8]

In retrospective studies, the incidence of Methotrexate-induced lung disease has been estimated to be 3.5–7.6%, with a prevalence of 5% [9], and has been shown to develop early during Methotrexate treatment, mostly within the first year [10].

Methotrexate can lead to multiple respiratory problems: hypersensitivity pneumonitis, interstitial fibrosis, acute lung injury with noncardiogenic pulmonary edema, organizing pneumonia, pleuritis and pleural effusions, and pulmonary nodules with acute interstitial hypersensitivity pneumonitis being the most common [11,12].

Methotrexate-induced pneumonitis is reported in about 0.43% to 1% of treated patients [10] with a mortality rate of 20% [13].

The risk factors for methotrexate-induced lung injury in RA patients are age (>60 years), previous DMARDs use (LEF, SSZ, gold, or D-penicillamine), preexistent respiratory manifestations of RA, hypoalbuminemia, diabetes, and renal dysfunction [11].

Sathi et al. adds as an additional risk factor for Methotrexate pneumonitis reduced baseline pulmonary function tests (PFT), advising baseline pulmonary function tests when initiating Methotrexate therapy [14]. However, the role of Methotrexate in the development of ILD in RA patients is debatable since the severity of RA is an independent risk factor for developing ILD [9,14].

In a meta-analysis of randomized controlled trials, Conway et al. demonstrated a slightly increased risk of respiratory adverse events, including ILD exacerbations in patients with RA treated with Methotrexate compared with other DMARDs and biologic agents (RR 1.10, 95% CI 1.02–1.19). However, the patients treated with Methotrexate did not have an increased risk of death due to pulmonary disease [9]. 

Methotrexate pneumonitis typically has an acute/subacute onset and often presents a hypersensitivity pneumonitis pattern [15]. Several days/weeks after initiating Methotrexate (or more gradually in the subacute form), the patient presents dyspnea, non-productive cough, fever, and, in some cases, acute respiratory failure [15,16].

Allergic mechanisms are thought to cause DLI because respiratory symptoms are often accompanied by fever and peripheral eosinophilia. Drug-induced lung injury most frequently shows widespread ground glass opacity (GGO) and hypersensitivity pneumonitis-like pattern, which may resemble the imaging findings from pneumocystis pneumonia (PCP) [15].

For a better diagnostic approach there have been developed diagnosis criteria for adverse pulmonary events associated with Methrotrexate use (Table 4).

The topic of chronic pulmonary injury induced by Methotrexate is highly debated: when investigating the chronic pulmonary effects of low-dose Methotrexate in RA patients (55 RA patients treated with Methotrexate and 75 RA patients in the control group), Dawson et al. did not find any evidence that Methotrexate leads to chronic pulmonary fibrosis. Moreover, in this study, it should be noted that during the 2-year follow-up, even though there was a deterioration in the pulmonary function parameters, this did not reach a significant difference [18].

In case of Methotrexate-induced ILD, treatment consists of drug cessation and, (especially in patients who remain symptomatic after Methotrexate withdrawal) corticosteroids. It is not advised to reinitiate Methotrexate after an ILD-event [11].

### 2.2. Leflunomide 

Leflunomide acts by inhibiting the mitochondrial enzyme dihydro-orotate dehydrogenase (DHODH) via its metabolite A771726—teriflunomide [19].

Leflunomide-induced/exacerbated ILD first became a concern in 2004, when, months after the drug was released in Japan, post-marketing surveillance showed that sixteen patients developed de novo or exacerbated ILD, nine of whom died [19].

There is confounding data given the fact that Leflunomide is often a second-line therapy and most patients receiving it have a Methotrexate history. A study by Suissa et al. showed that while Leflunomide use was a risk factor for developing RA-ILD, the patients at risk had either previously used Methotrexate or had a history of ILD. Moreover, the same study noticed a prescription bias: patients with preexistent ILD were twice as likely to be prescribed Leflunomide instead of Methotrexate. Therefore, in many cases, it remains difficult to demonstrate causality when suspecting Leflunomide-induced ILD [20]. 

The main pathogenetic mechanism of Leflunomide-induced pneumonitis is attributed to hypersensitivity reactions [1].

Racial factors seem to have a part in Leflunomide-induced ILD: Sawada et al. reported that, in a cohort of 5054 RA patients who were prescribed LEF, 61 patients (1.2%) developed ILD as an adverse reaction to this DMARD, which was higher than that estimated at 0.02% in Western countries [21]. Similarly, Ju et al. reported a higher prevalence of Leflunomide-induced ILD: 1.0% of the Korean patients treated with Leflunomide developed this adverse reaction [22]. The frequency of leflunomide-induced ILD and its associated mortality rate are greater compared to Western countries, as indicated by this data; however, the underlying mechanisms responsible for this racial disparity in susceptibility to drug-induced ILD remains unidentified.

It has been postulated that mechanisms connected to epithelial–mesenchymal transition (EMT) might be involved in Leflunomide-induced ILD [23]. In ILD, epithelial-mesenchymal transition (EMT) might contribute to the pulmonary build-up of fibroblasts and myofibroblasts, originating from epithelial cells.

Namba’s et al. research on Leflunomide’s metabolite, A771726, indicated that A771726 induced EMT-like characteristics in cultured human type II alveolar (A549) cells, via DHODH inhibition. A771726 treatment resulted in the upregulation of α-smooth muscle actin (α-SMA) and Col1a1 mRNA expression, and a downregulation in the expression of E-cadherin mRNA [23].

Leflunomide-induced pneumopathy usually occurs within the first 20 weeks of therapy, being typically described as being a NSIP pattern. Risk factors associated with such lung involvement include preexisting pulmonary lesions, interstitial pneumonia, the use of a loading dose, smoking, and low body weight [21,24].

### 2.3. Sulfasalazine, Hydroxychloroquineazathioprine, and Cyclosporine

Sulfasalazine-induced lung disease is rather rare. A retrospective case study involving all patients in published reports between 1972–1999 treated with sulfasalazine and who had developed a possible pulmonary adverse reaction to the drug identified only 50 patients. In this cohort, there were only six patients with RA [25].

There is currently no available data on the pulmonary toxicity of hydroxychloroquine.

Whilst there have been case reports on pulmonary toxicity in azathioprine-treated patients [26], azathioprine has been successfully used in RA-ILD (mostly via extrapolation from the therapeutic approach in ILD secondary to the other connective tissue disorders) [27]. In a retrospective cohort analysis that studied the combined incidence rate of death, transplant and respiratory hospitalization associated with azathioprine exposure when compared with mycophenolate mofetil demonstrated a marginally better response in regard to pulmonary function tests for the azathioprine group, but with a higher rate of side effects [28]. There have been multiple case reports in which cyclosporine has been associated with sustained pulmonary function improvement as well as rheumatoid arthritis control [29,30,31].

## 3. Biologic Agents (bDMARDs)

Most bDMARDs have been linked to lung toxicity, but they have also been reported to have the potential to improve lung function and stabilize pulmonary symptoms.

Regarding biological disease-modifying antirheumatic drugs (DMARDs), tumor necrosis factor (TNF) alpha inhibitors are those with the most data on drug-induced ILD.

### 3.1. Anti-TNF Drugs

Initial concerns regarding drug-induced ILD in RA patients arose after anecdotal reports of serious exacerbations of respiratory disease following treatment with a TNF inhibitor (TNFi) in patients with pre-existing RA-ILD. Post-marketing studies have revealed that the development of ILD after TNF inhibitor therapy was a rather rare event (0.5–0.6%), but that the preexisting ILD at the moment of TNF inhibitors initiation is a risk factor for ILD exacerbations [32,33,34]

When analyzing a RA cohort (163 patients with RA who underwent anti-TNF therapy), Nakashita et al. demonstrated a potential risk of ILD events (progression) in patients with pre-existing ILD: 24.1% of the patients with pre-existing ILD had subsequent ILD events, whilst only 3% of the patients without pre-existing ILD registered such events (still a higher proportion than the one reported by the post-marketing studies). This data, in conjunction with descriptions regarding each of the TNF alpha inhibitors, indicates that the risk of pneumonitis seems to display a class effect [34].

In a cohort of 122 RA patients with interstitial lung disease either induced or exacerbated by TNF-targeted therapies, complete resolution was observed in up to 40% after withdrawal of the biologic agent [35].

The data available from the British Society for Rheumatology Biologics Register showed that the survival of RA-ILD patients is not influenced by anti-TNF-α therapy [36].

The imaging pattern in TNF-inhibitors-induced ILD is variable with different patterns of interstitial involvement, most commonly UIP or NSIP; cases of organizing pneumonia, diffuse alveolar damage, and lymphoid interstitial pneumonia have also been described [37].

It should also be noted that patients with RA initiated on TNF inhibitors often continue conventional DMARD therapy. In patients with concomitant Methotrexate therapy, an increased frequency of development of Methotrexate pneumonitis has been suggested [38]. 

Regarding the relationship between specific biological medication and drug-induced ILD, post-marketing surveillance of infliximab’s safety profile in 5000 Japanese RA patients identified ILD in 25 patients (0.5%), after a mean of 2.8 infusions of infliximab, with a mean number of days from the first infusion to ILD diagnosis of 76.8 days (36–153 days) [32].

Similar data exist regarding Adalimumab: the data published by the Japan College of Rheumatology, on 3000 RA patients treated with adalimumab, reported the occurrence of interstitial pneumonia in 0.6% of patients. Additional post-marketing surveillance data on a cohort of 7440 patients placed the prevalence of ILD among adalimumab-treated patients at 0.5% [39,40].

Etanercept (ETN) was studied in a randomized controlled trial in the treatment of idiopathic pulmonary fibrosis, which showed no significant differences observed in the efficacy endpoints between the placebo and active treatment groups [41].

The post-marketing surveillance of etanercept in RA treatment, conducted by JCR (the Japan College of Rheumatology), revealed 0.6% of study patients developed ILD [33,42].

As to the impact of etanercept in combination with DMARDs, not only did the concomitant use of etanercept and methotrexate show a better RA control, but also significantly lower incidence rates for total adverse events, including ILD (when compared to etanercept as a monotherapy or associated with other DMARDs) [43]

Certolizumab, similarly to other TNFα inhibitors, may lead to acute exacerbations in RA-ILD patients, probably via NLRP3 inflammasome activation precipitate pneumonitis. The first case was reported in 2013 by Glaspole et al. [44,45] and further case reports have focused on new-onset or acute exacerbation (AE) of ILD in patients, but so far the data is limited [46].

### 3.2. Alternate Mechanisms of Action (MOAs) Agents

There is even less data regarding alternate mechanisms of action (MOAs) agents: T-cell, B-cell, and interleukin-6 inhibitors. 

A study by Curtis et al., evaluating ILD incidence and exacerbation among RA patients treated with MOAs (abatacept, rituximab, and tocilizumab) compared with anti-TNFα agents, showed no significant differences regarding the risk of ILD and its related complications. It should be also noted that the patients in the MOAs group were more likely to have prior exposure to other drugs; for instance, prior biologic exposure and corticosteroid use was highest in patients in the tocilizumab and rituximab groups [47].

### 3.3. Rituximab

Rituximab seems to be a relatively safe therapy in RA patients with lung involvement. Given the fact that a worsening of preexisting ILD in RA patients treated with TNF inhibitors has been reported in the literature, Rituximab has often been the biological agent of choice in these cases. Even though data available on the matter are still rather scarce, several studies have seemed to suggest that Rituximab has a better safety profile in RA-ILD patients 

In a cohort of 264 patients with RA that had received RTX, out of which 38 patients (14%) had lung involvement, lung disease remained clinically and radiologically stable in most patients, with just a single patient showing slow progression of the disease over 4 years of follow-up [48].

A cohort study involving 53 RA patients with preexisting ILD suggests that, at the very least, patients with stable ILD remain stable in regards to pulmonary function after the initiation of Rituximab therapy during prolonged follow-up [49].

Another small-scale study, which did not specifically focus on RA patients but rather on patients with ILD that did not respond to conventional immunosuppression, followed 44 patients initiated on Rituximab for a period of two years. The results of this study seem to indicate that Rituximab treatment is associated with a reduction in FVC and DLCO decline, especially when used in patients with connective-tissue-disease-associated ILD [50].

Similar results were published by Franzen et al. In a cohort of 33 RA patients treated with Rituximab, when evaluated with serial pulmonary function tests, while no instances of respiratory symptoms were reported, the DLCO showed a progressive decline during follow-up with a maximum reduction of 6.1% at 26 weeks (when compared to baseline). The risk factors for pulmonary function changes post-rituximab were cigarette smoking, repeated administration of the drug, and corticosteroid use (prednisone). The gradual decrease in DLCO suggests the possibility of subclinical pulmonary toxicity induced by rituximab, but it should be noted that both steroid use and repeated courses of rituximab are linked to a more severe RA [51].

In regards to patient mortality, the data from the British Society for Rheumatology Biologics Register for RA (BSRBR-RA) suggest that the RA-ILD who were administered rituximab as their initial biologic had superior long-term survival rates when compared to those who began treatment with TNF inhibitors, with an adjusted 5-year risk of mortality in the RTX-treated patients of approximately half that in the TNF inhibitor-treated patients, but the difference was not statistically significant (HR 0.53, 95% CI: 0.26 to 1.10) [52].

### 3.4. Abatacept

At the time of this review, the data on the impact of Abatacept in patients with RA-ILD are reassuring. A multicenter study including 263 RA-ILD patients treated with Abatacept showed an improvement or stabilization after 1 year of therapy regarding dyspnea scores (in 91.9%), FVC (in 87.7%), DLCO (in 90.6%), and chest HRCT (in 76.6%) [53].

A prospective study on a cohort of 57 RA-ILD patients treated with abatacept for a median (IQR) of 27.3 (12.2–42.8) months demonstrated both arthritis control and pulmonary involvement stabilization in 71% of patients. The factors associated with lung disease progression and mortality were high disease activity (calculated with the DAS28-ESR formula) and low baseline DLCO and FVC values [54].

In another cohort of 44 patients with RA-ILD treated with Abatacept, only 11.4% experienced a significant worsening during the 18-month follow-up period (when evaluated on the change in the percentage of fibrosis). The variables associated with RA-ILD worsening were current smoker status and concomitant Methotrexate use [55]. 

Another study that compared the effect of biological therapies on airway and interstitial lung disease in RA patients found abatacept to be an independent protective factor for both RA-ILD, but also for airway disease exacerbation [56]. 

When comparing the safety of Abatacept in monotherapy with ABA associated with synthetic DMARDs in RA patients with interstitial lung disease, both strategies seem to have similar safety and effectiveness profiles. In a retrospective multicenter study of RA-ILD Caucasian patients treated with abatacept either as monotherapy associated with Methotrexate or non-Methotrexate DMARDs, more than 70% (out of 263) of the patients had stable or improved ILD after 18 months of treatment, with no significant differences between treatment groups [57].

### 3.5. Tocilizumab

There are data suggesting that uncontrolled arthritis activity during tocilizumab treatment could lead to an acute exacerbation of RA-ILD. A retrospective, case-controlled study involved 395 consecutive RA patients treated with tocilizumab, 78 with ILD, and 317 without ILD. Six acute exacerbations occurred, after a median treatment duration of 48 weeks. The patients experiencing acute ILD exacerbations had higher disease activity measured using the Clinical Disease Activity Index (CDAI) at 24 weeks (20.8 vs. 6.2, *p* = 0.019), suggesting that the acute exacerbations of RA-ILD were likely attributable to uncontrolled disease activity rather than an adverse effect of the drug [58]

In the post-marketing data of a cohort of 7901 RA patients, with a cumulative exposure to tocilizumab of 3831.8 patient-years (PY), 38 (0.5%, with an incidence rate of 1.0 event per 100 person-years) were reported to have interstitial lung disease (ILD), with 22 of them having either a concurrent or prior medical history of ILD at the beginning of the study. It should be noted also that 24 of the ILD patients had a prior history of biological DMARDs. Multivariate logistic regression analysis identified advanced age (≥65 years) and previous or concurrent ILD at baseline as the risk factors for ILD [59].

In a study that included 125 elderly (>65 years) patients with RA treated either with Abatacept (n = 47) or Tocilizumab (n = 78), the most common adverse event that resulted in the discontinuation of Tocilizumab treatment was ILD. Out of the five patients who developed ILD while on Tocilizumab, two had a worsening of pre-existing ILD, while four had been concurrently using Methotrexate. In contrast, ILD was not reported as an adverse event leading to the discontinuation of abatacept in elderly patients with RA [60].

In a cohort of 11,219 patients, totaling 13,795 episodes of biologic exposure, there were no significant differences in the risk of ILD and its related complications between RA patients receiving different classes of biological therapy. The data suggests that the incidence and exacerbation of ILD in the Tocilizumab and Rituximab groups may be exaggerated by treatment resistance or by the severity of the disease, since the first line of biologics were anti-TNF agents in most patients [47].

### 3.6. Anakinra

Information regarding anakinra’s pulmonary effects in RA patients is scarce, and so far, there have not been any randomized controlled trials in regard to RA-ILD. The data scarcity may be because anakinra use has seen a significant decline over time. The newer therapeutic options seem to provide better RA control, a better safety profile, and more convenient dosing options.

## 4. Targeted-Synthetic DMARDS/JANUS KINASE Inhibitors

Regarding targeted-synthetic DMARDs, which represent a somewhat newer alternative in RA treatment, information pertaining to interstitial lung disease and pulmonary safety is currently limited, but the results published thus far seem to indicate a low rate of ILD development during Janus-kinase inhibitor use [61,62,63].

Moreover, there is data regarding Tofacitinib: a post hoc analysis of 21 trials, including 7061 patients (patient-years of exposure 23,393.7) who received tofacitinib, showed that the IR for an ILD event was 0.18 for both tofacitinib 5 mg BID and 10 mg BID, an incidence associated with known risk factors of RA-ILD, such as age, smoker status, and high disease activity (DAS28 scores) [62].

A retrospective study using claims data pertaining to 28,559 patients with RA from the Optum Clinformatics Data Mart Database showed that, when calculating the IRs per 1000 person-years for ILD, patients who received tofacitinib were 69% less likely to develop ILD (IR = 1.48) compared to those treated with adalimumab (IR = 4.30) [63].

In a retrospective study of 75 patients with RA and ILD treated either with Janus kinase inhibitors (tofacitinib 5 mg BID or baricitinib 4 mg daily) or abatacept (125 mg/week) for at least 18 months, the data showed similar safety profiles in regard to pulmonary outcomes. RA-ILD stability or improvement was reached in 83.9% and 88.6% of patients, respectively. In the multivariate regression analysis, the only variable related to RA-ILD deterioration in patients treated with Janus kinase inhibitors was disease duration (*p*  <  0.001) [64].

In a descriptive, multicentric, retrospective cohort study that included data pooled from eight randomized trials, with 3770 RA patients treated with baricitinib, with 12,358 patient-years of exposure, Salvarani et al. identified 21 ILD cases with an exposure-adjusted incidence rate (EAIR) of 0.17 per 100 patient-years of exposure (PYE), proving a low risk of developing ILD during treatment [65].

Furthermore, in a retrospective exploratory study that analyzed a cohort of 43 RA-ILD patients treated with either baricitinib (65.12%), tofacitinib (20.93%), filgotinib (6.98%), or upadacitinib (6.98%), the DLCO improved or remained stable in 80% of the cases. The forced vital capacity followed the same trend, as it worsened in only 10.71% of the patients and the chest HRCT demonstrated a progression of ILD in 9.30%. While combined therapy with methotrexate was documented in 38.10% of the patients, no improvement or deterioration was seen in the HRCT and pulmonary function tests between those with monotherapy or those with concomitant methotrexate use [66]. 

These results suggest that treatment with Janus kinase inhibitors may provide benefit in reducing the risk of developing RA-ILD. 

## 5. Conclusions and Research Agenda

The establishment of treatment guidelines for RA-ILD continues to present an ongoing challenge, with further complications arising from the potential pulmonary toxicity of both synthetic and biologic DMARDs. Our review will hopefully be able to aid the clinician in choosing the optimal treatment strategy for both at-risk patients and those with established ILD.

Both initial and subsequent evaluations of RA patients should consider both articular and extraarticular manifestations. Risk factors (such as smoking or environmental exposures), clinical findings, dyspnea scales, the 6-min walk test, pulmonary function tests (PFT), DLCO, and imaging findings should be documented in order to assess the severity of pulmonary involvement and to detect its progression.

Routine clinical evaluations of RA patients should include pulmonary evaluation, with an emphasis on a structured follow-up plan for individuals with identified ILD or those at high risk, including regular clinical evaluations, PFTs, and imaging assessment.

Optimizing therapy and follow-up strategies require a multidisciplinary approach that involves, at a minimum, a rheumatologist, a pulmonologist, and a radiologist. Drug-induced lung damage should also be considered in any RA patient who develops respiratory symptoms or new imaging changes during therapy.

In patients with low disease activity/remission and with stable pulmonary involvement, the ongoing therapy should be continued; however, in patients with progressive ILD and active joint disease, switching to JAK inhibitors or alternate mechanisms of action (MOAs) agents could be beneficial.

During the past two decades, we have witnessed many exciting developments in the treatment of rheumatoid arthritis. While this is very encouraging, ongoing research regarding the long-term safety profile of these agents is essential, especially among patients with extra-articular manifestations such as RA-ILD, or patients with preexisting pulmonary conditions. Moreover, there is a need for clinical registries/randomized controlled trials on antifibrotic agents and DMARD associations.

## Figures and Tables

**Table 1:** Diagnostic criteria for drug-induced lung injury [3].

History of Ingestion of a Drug that is Known to Induce Lung Injury	Specifically Inquire about the Following when Taking the Patient’s History: Over-the-Counter (OTC) Drugs, Health Foods, and Illegal Narcotic Drugs/Anti-Hypnotic Drugs
The clinical manifestation has been reported to be induced by a drug	The clinical manifestations include clinical findings, imaging findings, and pathological features.
Other causes of the clinical manifestation could be ruled out	Differentiation from infection, cardiogenic pulmonary edema, exacerbation of an underlying disease, etc.
Improvement of the clinical manifestations after drug discontinuation	Spontaneous remission or remission in response to a corticosteroid.
Exacerbation of the clinical manifestations after resuming drug administration	Resuming drug administration to identify the causative drug is not generally recommended but is acceptable if the patient requires the drug and safety is assured.

**Table 2:** Pathogenetic mechanisms of drug-induced lung injury.

Cytotoxic effects (e.g., methotrexate, cyclophosphamide, sulfasalazine)	Direct damage to alveolar epithelium and endothelialium. Mediated by reactive oxygen species, proteases, and cytokines. Pulmonary fibrosis due to increased vascular permeability, inflammation, and tissue injury (extent of injury is generally usually dose and duration dependent).
Activation of immune cells (allergic reaction) (e.g., anti TNF alpha)	Immunogenicity due to drug/drugmetabolite binding to cytoplasmic proteins (hapten hypothesis). Eosinophil infiltration in the alveolar wall and airspaces (eosinophilic pneumonia). Interstitial pneumonia mediated by lymphocyte infiltration and granuloma formation in the alveolar wall (involved in most cases of DLI and usually neither dose nor duration related).
Host factors	Age, smoker status, exposure to chemicals/dusts, genetic factors. Underlying pulmonary diseases: pulmonary fibrosis, chronic obstructive pulmonary disease (COPD), and emphysema. Iatrogenic: exposure to high concentrations of oxygen, history of radiation exposure, surgery

Adapted from [1].

**Table 3:** DMARDs used in RA treatment [8].

Synthetic DMARDs	Conventional—csDMARDs	Methotrexate, Leflunomide, Hydroxychloroquine, Sulfasalazine, Cyclosporine, Azathioprine
	Targeted—tsDMARDs/JAK Inhibitors	Tofacitinib, Baricitinib, Upadacitinib,
Biological DMARDs	TNF Alpha inhibitors	Infliximab, Etanercept, Adalimumab, Golimumab, Certolizumab
	CD 20 receptors on B cells inhibitor	Rituximab
	Interleukin 6 receptor antagonist	Tocilizumab
	Selective T cell co-stimulation modulator	Abatacept
	Interleukin 1 receptor antagonist	Anakinra

**Table 4:** Searles and McKendry diagnostic criteria for adverse pulmonary events associated with methotrexate treatment in rheumatoid arthritis [16,17].

**Major criteria**	I. Hypersensitivity pneumonitis (demonstrated by histopathologic examination, with no evidence of pathogenic organisms).
	2. Radiologic evidence of pulmonary interstitial or alveolar infiltrates.
	3. Negative blood (if afebrile) and initial sputum (if productive) cultures.
**Minor criteria**	1. Shortness of breath of <8 weeks duration.
	2. Non-productive cough.
	3. O2 saturation < 90% at the time of initial evaluation.
	4. DLCO < 70% of the predicted value
	5. WBC ≤ 15,000 per mm^3^.
Definite cases were defined as the presence of major criterion 1 or major criteria 2 and 3, and three out of the five minor criteria. Probable cases were defined as the presence of major criteria 2 and 3, and two minor criteria. DLCO = diffusing capacity for carbon monoxide; WBC = white blood cell (count).	

Adapted from [16,17].

## Data Availability

Not applicable.
